# Host–Pathogen Interactions and Correlated Factors That Are Affected in Replicative-Aged *Cryptococcus neoformans*

**DOI:** 10.3390/jof10040279

**Published:** 2024-04-10

**Authors:** Vanessa K. A. Silva, Sungyun Min, Kyungyoon Yoo, Bettina C. Fries

**Affiliations:** 1 Division of Infectious Diseases, Department of Medicine, Stony Brook University, Stony Brook, NY 11794, USA; vanessakarina.alvesdasilva@stonybrookmedicine.edu (V.K.A.S.); smin2027@nycpm.edu (S.M.); 2 Department of Microbiology and Immunology, Renaissance School of Medicine, Stony Brook University, Stony Brook, NY 11794, USA; kyungyoon.yoo@stonybrookmedicine.edu; 3 Veterans Administration Medical Center, Northport, NY 11768, USA

**Keywords:** aging, cryptococcosis, G2 arrest, intracellular parasitism, phagosomal pH, polysaccharide capsule, vomocytosis, urease

## Abstract

*Cryptococcus neoformans* is a facultative intracellular fungal pathogen. Ten-generation-old (10GEN) *C. neoformans* cells are more resistant to phagocytosis and killing by macrophages than younger daughter cells. However, mechanisms that mediate this resistance and intracellular parasitism are poorly understood. Here, we identified important factors for the intracellular survival of 10GEN *C. neoformans*, such as urease activity, capsule synthesis, and DNA content using flow cytometry and fluorescent microscopy techniques. The real-time visualization of time-lapse imaging was applied to determine the phagosomal acidity, membrane permeability, and vomocytosis (non-lytic exocytosis) rate in J774 macrophages that phagocytosed *C. neoformans* of different generational ages. Our results showed that old *C. neoformans* exhibited higher urease activity and enhanced Golgi activity. In addition, old *C. neoformans* were more likely to be arrested in the G2 phase, resulting in the occasional formation of aberrant trimera-like cells. To finish, the advanced generational age of the yeast cells slightly reduced vomocytosis events within host cells, which might be associated with increased phagolysosome pH and membrane permeability. Altogether, our results suggest that old *C. neoformans* prevail within acidic phagolysosomes and can manipulate the phagosome pH. These strategies may be used by old *C. neoformans* to resist phagosomal killing and drive cryptococcosis pathogenesis. The comprehension of these essential host–pathogen interactions could further shed light on mechanisms that bring new insights for novel antifungal therapeutic design.

## 1. Introduction

Virulence strategies employed by the encapsulated yeast *Cryptococcus neoformans* encompass the capability to directly manipulate the host cell (Casadevall and Fang [1]). This facultative intracellular organism has adapted to reside and replicate within the acidic phagosome and to escape via a non-lytic process called vomocytosis [2]. All these mechanisms can be exploited by *C. neoformans* to transverse the lung epithelial tissues and travel in the blood circulation, disseminating as a ‘Trojan horse’ to extrapulmonary organs, including the brain [3].

*C. neoformans* is the leading cause of fungal meningitis [4]. In response to this public health threat, this fungal pathogen was recently ranked as critically important by the World Health Organization [5,6]. *C. neoformans* is a eukaryotic pathogen that undergoes asymmetric division during replication, resulting in a phenotypic dichotomy between the aging mother cell and its newly budded daughter [7]. Although labeled as ‘old’, these cells continue to exhibit fitness with unchanged doubling times, having lived only about a third of their life expectancy, and are far from senescence. *C. neoformans* cells that have lived for 10 generations exhibit marked resistance to phagocytic killing [8], increased melanin secretion [9], a remodeled cell wall [10], and a higher tolerance to antifungals [8]. We have proposed that the persistence of cryptococcal disease and treatment failure during chronic infection is driven by the selection and accumulation of old yeast cells, which can evade the host response [8].

The arsenal of virulent factors employed by *C. neoformans* to survive inside a host cell include the release of capsular material and the secretion of enzymes, such as urease [11,12]. For instance, the cryptococcal polysaccharide capsule [13] and unbudded cell cycle arrest [14] have been linked to a loss of phagosome membrane integrity, whereas urease activity affects fitness, increases phagosomal pH within the mammalian phagosome, and promotes vomocytosis [12].

Based on pivotal studies that characterized *C. neoformans* interactions with macrophages [15], we hypothesized that *C. neoformans* phagosome interactions impose selection on the *C. neoformans* population in the host. In this study, we investigated the strategies used by old *C. neoformans* to thrive inside host macrophages, and we characterized the age-associated virulence factors that contribute to fungal intracellular survival.

## 2. Materials and Methods

### 2.1. Yeast Strains and Isolation of Old C. neoformans Cells

*C. neoformans* wild-type strains (H99 and KN99α) were maintained on YPD agar plates. Strain KN99α was used for the urease activity test, and H99 was used for all the remaining experiments. Yeast cells (10^8^) from a six-hour exponential culture in synthetic media (1.7 g yeast nitrogen base without amino acids, 1 g drop-out mix, 0.4% ethanol, 5 g (NH_4_)_2_SO_4_, 3.3 g NaCl, 20 g glucose) at 37 °C and 160 rpm shaking were washed twice with phosphate-buffered saline (PBS) pH 7.0, and then labeled with 8 mg/mL Sulfo-NHS-LC-LC-Biotin (Thermo Fisher Scientific, Waltham, MA, USA) for 30 min at room temperature. Subsequently, *C. neoformans* cells were washed with PBS and grown for 5 doubling times (doubling times of *C. neoformans* range from 2.7 h to 5.2 h) [16] in the same exponential culture conditions. Afterwards, the yeast cultures were washed once with PBS containing 0.5 M Ethylenediaminetetraacetic acid (EDTA) for the removal of DNA from cell debris and to reduce cell clumping, and twice with PBS alone. Then, 100 μL of magnetic streptavidin microbeads were added to previously labeled (10^8^) cells in PBS, following incubation for 15 min at 4 °C. Subsequently, biotin-labeled yeasts were isolated using autoMACS magnetic columns and the autoMACS Pro separator (Miltenyi Biotec, Bergisch Gladbach, Germany). The biotin-streptavidin-labeled older cells were passed through a magnet where they were immobilized, while the younger unlabeled population flew through. The labeled cells were recovered after the magnetic field was removed. The positive cell fraction was grown again in a synthetic media until the desired generation was reached (10 GEN) and isolated as outlined above. Young cells washed off from the magnetic columns were kept as controls (adapted from [10]). 

### 2.2. Macrophage Cell Line Culture

Cells from the murine macrophage-like cell line J774A.1 were used between passages 4 and 14 following thawing, and were cultured in Dulbecco’s modified Eagle’s medium (DMEM) (Gibco, Life Technologies, Carlsbad, CA, USA) supplemented with 2 mM l-glutamine, 1% Sodium Pyruvate, 100 U/mL penicillin, 100 U/mL streptomycin, and 10% fetal bovine serum (FBS) at 37 °C and 5% CO_2_.

### 2.3. Urease Activity

Furthermore, 10^7^ *C. neoformans* cells were incubated in a rapid urea broth (Urea 4 g, Yeast extract 0.02 g, Phenol Red 2 mg, KH_2_PO_4_ 0.273 g, Na_2_HPO_4_ 0.285 g, H_2_O 100 mL) [17] for 3 h at 37 °C with agitation. The optical density (OD) was read (λ 560 nm), and an OD > 0.3 was considered positive. Young and old *C. neoformans* cells were heat-killed for 30 min at 75 °C [18] to be used as a negative control. 

### 2.4. Analysis of the Golgi Apparatus

After the isolation of old cells, we followed the protocol adapted for the analysis of the Golgi in cryptococci by Kmetzsch et al. (2011) [19] and Rizzo et al. (2014) [20]. Briefly, young and old yeast cells (10^7^) were fixed with 4% paraformaldehyde in PBS, followed by washing with PBS and incubation with C6-NBD-ceramide (10 μM) for 16 h at 4 °C. The cells were then incubated with fetal calf serum (10%) at 4 °C for 1 h to remove the excess of C6-NBD-ceramide. The cell wall was stained with calcofluor white (5 μg/mL) for 10 min at room temperature, followed by washing with PBS and analysis using fluorescence microscopy (Nikon Eclipse 90i microscope, Tokyo, Japan). Different staining patterns (central or peripherical) were determined in approximately 100 cells of each strain using the ImageJ software v.154g (NIH, Bethesda, MD, USA).

### 2.5. Polysaccharide Capsule Analysis

Following old cells’ separation from young fungal cells, both young and old yeast cells (10^7^) were fixed in 4% paraformaldehyde for 1 h and then incubated in PBS supplemented with 1% bovine serum albumin for 1 h at 37 °C. The samples were incubated with the monoclonal antibodies (mAb) 18B7 (IgG1) at 10 µg/mL for 1 h at 37 °C [21]. After washing with PBS, the *C. neoformans* cells were incubated with an anti-murine IgG Alexa Fluor™ 488-conjugated (Invitrogen, Waltham, MA, USA) at 10 µg/mL. Finally, the yeast cells were again washed, suspended in PBS, and then analyzed using flow cytometry and fluorescent microscopy [22].

The capsule of *C. neoformans* was analyzed on a BD LRSFortessa flow cytometer (Franklin Lakes, NJ, USA) with the blue laser (488 nm). Unstained cells were used as negative controls. A total of 10,000 events were gated in the forward scatter/side scatter (FSC/SSC) plots, which were then represented as histograms, with the mean fluorescence intensity (MFI) on the x-axis and the cell counts on the y-axis. The data were analyzed using FlowJo v10.8.1 software (BD Biosciences, Franklin Lakes, NJ, USA). For fluorescence microscopy, the fluorescein isothiocyanate (FITC) (DyLight 488) and DAPI (4′,6-diamidino-2-phenylindole) (CFW) channels were used. Imaging was performed at 100× magnification in a Nikon Eclipse 90i microscope (Tokyo, Japan) with a digital camera. The same exposure time was used to image young and old cells, and the images were processed using ImageJ software v.154g (NIH, Bethesda, MD, USA).

### 2.6. DNA Staining and Cell Cycle Analysis

We analyzed the DNA levels and cell cycle by staining *C. neoformans* cells with propidium iodide, according to a previous study [23]. Briefly, after standard magnetic bead-based isolation, 5 × 10^6^ cells of (young and old) *C. neoformans* H99 and diploid strain #K24 cells (positive control) were centrifugated at 500 RCF for 3 min, and then fixed with 500 µL of 70% ethanol overnight at 4 °C with rotation. The next day, the cells were centrifuged again at 500 RCF for 3 min, and the pellets were washed once in 500 µL of 50 mM sodium citrate. The cell suspensions were sonicated for 10–15 s at 30% power. The sonicated cells were centrifuged again at 500 RCF for 3 min, and the supernatants were gently aspirated and resuspended in 200 µL of 20 mM sodium citrate with 0.5 mg/mL RNAse and were then gently mixed. The samples were incubated at 37 °C for 2–4 h with rotation. Subsequently, 10 µL aliquots of each suspension were reserved to be used as unstained cells (negative control), and 5 µL of 50 mM sodium citrate and 5 µL of Propidium iodide (PI, 1 mg/mL) were added to the rest of the cell suspensions, resulting in a final concentration of 25 µg/mL per sample. Following incubation overnight in the dark at 37 °C with rotation, the samples were sonicated for 5–10 s at 15% of total power immediately before analyzing on the flow cytometer in order to dissociate any cell clumps. To avoid dye leaching out of the cells, the samples were diluted 1/40 into the PI buffer (25 µg/mL PI in 50 mM sodium citrate). To ensure statistical power in the analysis, at least 20,000 events per sample were acquired using a BD LRSFortessa flow cytometer (Franklin Lakes, NJ, USA) with a blue laser (488 nm) and the PE channel. 

### 2.7. Phagocytosis Assay, Phagosomal Acidification, and Phagosomal Permeability

J774A.1 macrophages (10^5^ cells/well) were seeded into a 24-well plate for 18 h, following activation with phorbol 12-myristate 13-acetate (PMA) (15 ng/mL) for 1 h. As controls, yeasts (2.5 × 10^6^ cells) were heat-killed at 60 °C for 1 h before opsonization with 10% human serum [24].

To analyze the pH alterations in the phagosomes infected with young and old *C. neoformans*, we used the amine-reactive pH dye Phrodo green^AM^ (P35373, Thermo Fisher Scientific, Waltham, MA, USA) to label the surface of *C. neoformans*, according to the manufacturer’s instructions and adapted from [15,25]. This staining is non-fluorescent at a neutral pH, but then becomes fluorescent at an acidic pH. *C. neoformans* young and old cells (2.5 × 10^6^ cells) were labeled with 500 μL of a solution containing 5 μL of pHrodo staining, 50 μL of Power load concentrate, and 5 mL of PBS. The yeast cells were incubated in the dark for 30 min at 37 °C with rotation. Following this, yeast cells were pelleted and resuspended in DMEM (Gibco, Life Technologies, Carlsbad, CA, USA) supplemented with 2 mM l-glutamine, 1% Sodium Pyruvate, 100 U/mL penicillin, and 100 U/mL streptomycin. Macrophages were then infected for two hours with serum-opsonized *C. neoformans* (MOI 10:1) at 37 °C and 5% CO_2_. After phagocytosis, the wells were washed with PBS at least three times to remove extracellular *C. neoformans*. Based on previous evidence, the phagosomal acidification behaviors in *C. neoformans* were classified as follows: 1. acidic; 2. delayed acidification; or 3. non-acidic [15,24].

Alternatively, two hours post-infection, the medium was replaced with serum-free DMEM (Gibco, Life Technologies, Carlsbad, CA, USA) supplemented with 50 nM LysoTracker^®^ Red DND-99 to check the phagosomal permeability. Yeast cells were then taken for time-lapse microscopy as described [15,24].

### 2.8. Time-Lapse Microscopy and Vomocytosis Rate

Time-lapse movies were made using a Nikon Ti2-E PFS with Live-Cell Imaging and a Tokai Hit Enclosure Incubator with Gas Mixing. Samples were incubated at 37 °C and 5% CO_2_ in the microscope imaging chamber. Images were taken every 5 min for 2 h and compiled into single movie files for analysis using NIS Elements Viewer v 5.21 (Prague, Czech Republic) or ImageJ software v.154g (NIH, Bethesda, MD, USA), respectively. Movies were blinded by a third party before manual scoring for vomocytosis and phagosome acidity. Macrophages infected with *C. neoformans* containing at least one acidic phagolysosome were counted as acidic. Vomocytosis was scored visually using the following previously established guidelines [2]: (a) one vomocytosis event is the expulsion of internalized cryptococci from an infected macrophage, regardless of the number of cryptococci expelled, if they do so simultaneously; (b) vomocytosis events are scored as independent phenomena if they occur in different frames or from different macrophages; (c) vomocytosis events are discounted if the host macrophage subsequently undergoes lysis or apoptosis within 30 min. 

### 2.9. Data Analysis

The data were plotted using GraphPad Prism v9.5 (La Jolla, CA, USA) and statistically analyzed through comparing young and old groups, using Student *t*-test with post-Welch corrections. Statistical significance was observed when the *p*-value was inferior to 0.05. All experiments were completed in biological triplicates otherwise stated in the figure legends. Images and videos were processed using ImageJ software v.154g (NIH, Bethesda, MD, USA) or the NIS-Elements Viewer v 5.21 software (Prague, Czech Republic), and at least 100 cells were analyzed for each group; the specific analyses are described in the figure legends. The flow cytometry data were analyzed using the FlowJo software v10.8.1 (BD Biosciences, Franklin Lakes, NJ, USA), and doublets were excluded using the following gating strategy of creating a scatterplot of PI (area) by PI (width). Single cells fell in a vertical line along the PI (width) axis. Cells were then gated in order to exclude auto-fluorescence using unstained control cells. Cell cycle analyses were performed using the Dean-Jet-Fox algorithm.

## 3. Results

### 3.1. Effects of Aging on Urease Activity in C. neoformans

**Figure 1:**
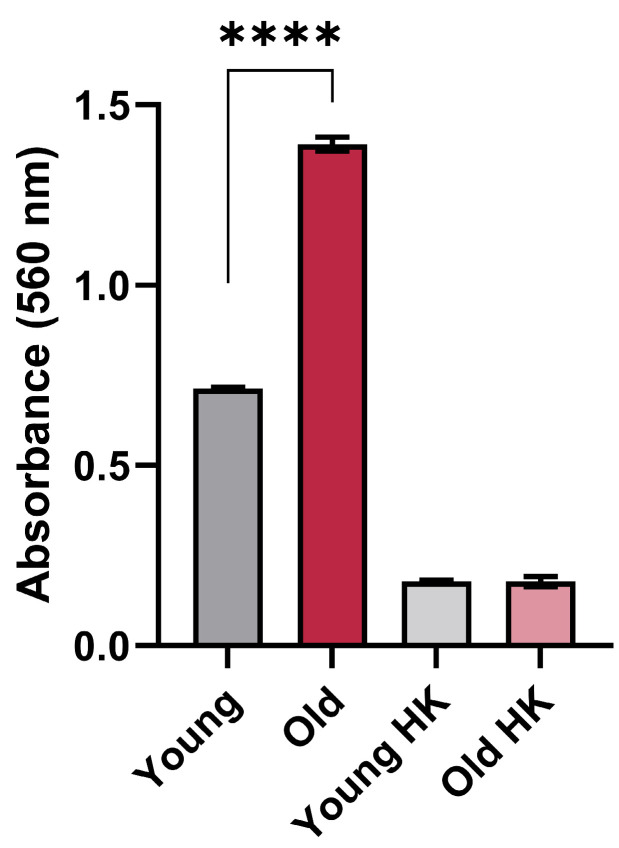
Urease activity in young and old *C. neoformans* KN99α strain. Urease enzymatic activity levels are increased in old *C. neoformans* (KN99α) cells in comparison with young *C. neoformans* (KN99α) (****, *p* < 0.001). Yeast cells were incubated in a rapid urea broth for 3 h at 37 °C with agitation, and the optical density (OD) was read (λ 560 nm). OD > 0.3 was considered positive, and heat-killed (HK) cells were used as a negative control. The graph represents one biological triplicate of three independent experiments with similar results. Statistical analysis was performed with unpaired *t*-test and post-Welch’s correction. Error bars represent the standard deviation between technical replicates.

Urease-mediated ammonia can neutralize acidic microenvironments, helping pathogens to survive the hostile pH of the phagolysosome [12]. We therefore tested if old *C. neoformans* produce more urease than young cells. For this, we analyzed the urease activity of young and old *C. neoformans*. The quantification of the urease activity of old *C. neoformans* remarkably increased in comparison to young *C. neoformans* (*p* < 0.001) (Figure 1).

This suggests that old *C. neoformans* cells exhibit superior abilities to hydrolyze urea than young *C. neoformans* cells.

### 3.2. Longevity in C. neoformans Affects Golgi Apparatus and Capsular Properties 

**Figure 2:**
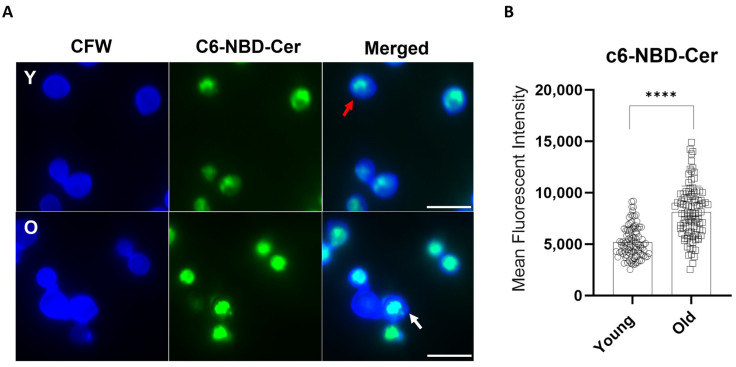
Influence of **the** replicative aging of H99 *C. neoformans* in the Golgi apparatus morphological aspects. (**A**) Young (Y) and old (O) *C. neoformans* cells were labeled with C6-NBD-ceramide (green), and the cell wall was stained with calcofluor white (blue). Peripherical (red) or central (white) Golgi morphologies are indicated by the arrows. Scale bars represent 10 µm. Images were taken using a fluorescent microscope at 100× and were analyzed using ImageJ software v.154g (NIH, Bethesda, MD, USA). (**B**) The quantitative analysis of the morphological profiles that predominated in young and old yeast cells. At least 100 cells per group (young and old) were analyzed, and the statistical analyses were performed using unpaired *t*-test with post-Welch’s correction (**** *p* < 0.0001).

Since the Golgi secretion is associated with the release of virulence factors, including the GXM synthesis [26], and this polysaccharide can also affect the fate of the yeast within the host cell [11], we next investigated the impact of generational age on Golgi aspects and capsular binding to mAb anti-GXM. Prior quantitative analysis through immunogold labeling had detected a significant increase in intracellular GXM associated with vesicular structures, vacuoles, and in the cell wall in old *C. neoformans* cells when compared to young *C. neoformans* cells [10]. Since GXM is synthesized in the Golgi complex [26], we used the Golgi marker N-[7-(4-nitrobenzo-2-oxa-1,3-diazole)]-6-aminocaproyl-D-erythro-sphingosine (C6-NBD-ceramide) to evaluate the morphological aspects and distribution of the Golgi apparatus in young and old *C. neoformans* cells (Figure 2A). The peripherical pattern was predominant both in young (66.85%) and old (60%) *C. neoformans* cells (Table 1). However, in the old *C. neoformans*, Golgi staining was significantly more intense when compared to young cells (MFI levels: 5184.91 vs. 8115.95) (Figure 2B). These results suggested an increase in the size of the Golgi apparatus in *C. neoformans* cells of advanced generational age. 

**Figure 3:**
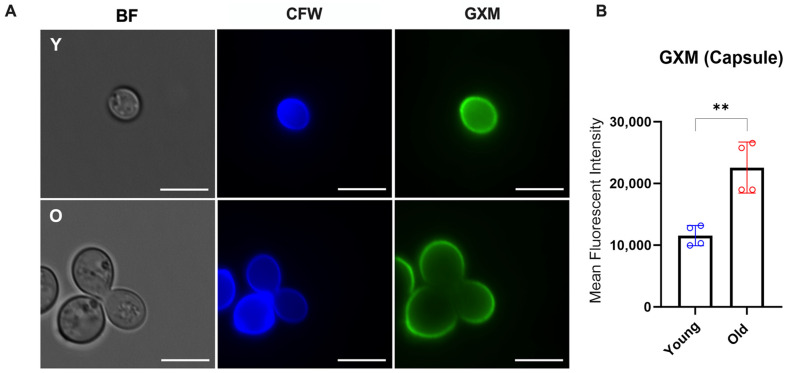
Morphological capsule analysis of H99 *C. neoformans*. (**A**) Representative images of young (Y) and old (O) yeast cells observed by fluorescence microscopy (GXM: green; Cell wall chitin: blue). Stained cells were imaged using a fluorescent microscope at 100× magnification and the following channels: DAPI (CFW, calcofluor white and GFP (GXM, 18B7-IgG Alexa Fluor 488), BF (brightfield). The scales bar represents 10 µm. (**B**) Flow cytometry to quantify the binding of capsular anti-GXM levels for old (red) and young (blue) *C. neoformans* (**, *p* < 0.01). Statistical analysis was performed using a *t*-test with Welch’s correction from two biological replicates.

Next, we compared the staining patterns of the polysaccharide capsule using fluorescent microscopy and flow cytometry following the staining with the GXM-specific monoclonal antibody 18B7. Although antibody binding patterns on the capsule architecture were similar in young and old *C. neoformans* (Figure 3A), the quantitative levels of mAb 18b7 binding to the capsular GXM were significantly higher in old *C. neoformans* cells than in young cells (**, *p* = 0.0082) (Figure 3B). These data suggest that old *C. neoformans* have enhanced Golgi activity and increased binding properties to the anti-GXM mAb on the capsule when compared to young cells. 

### 3.3. Old C. neoformans Presented an Increase in DNA Content and G2 Arrest

Prolonged cell cycle progression leads to *C. neoformans* cells with larger capsules, which were also associated with reduced phagocytosis and enhanced intracellular survival [27]. Since old *C. neoformans* exhibited increased binding of polysaccharide mAb 18B7, impaired phagocytosis, and enhanced intracellular survival [7,8,9], we investigated if old *C. neoformans* cells had altered DNA content indicative of a specific cell cycle state.

**Figure 4:**
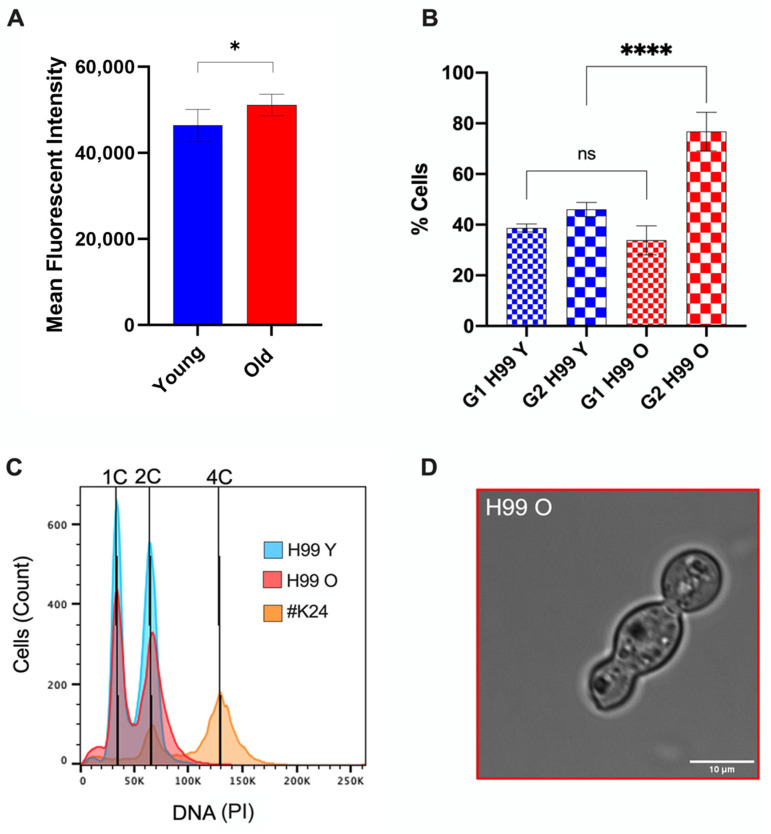
Old generation H99 *C. neoformans* cells presented G2 arrest. (**A**) *C. neoformans* cells were fixed, stained with propidium iodide, and analyzed using flow cytometry for the DNA content levels of young (blue) and old (red) *C. neoformans*. (**B**) Percentages of G1 and G2 phases in *C. neoformans* young (blue) and old (red) yeast cells. Unpaired *t*-test with Welch’s correction was performed to compare young and the old groups (* *p* < 0.01, **** *p* < 0.0001, and ns: nonsignificant *p* = 0.2908). (**C**) Representative histograms to determine cell ploidies within the population, showing peaks for *C. neoformans* cells of wild-type strains: young in blue, old in red (O), and the control diploid *C. neoformans* strain (#K24) in orange. (**D**) Brightfield image of the atypical elongated old *C. neoformans* cell, resembling a trimera-like formation.

To determine the DNA content of young and old *C. neoformans*, yeast cells were fixed, stained with PI, and examined using fluorescence flow cytometry. DNA levels were estimated based on the fluorescence intensity. These data demonstrated a statistically significant overall increase in nuclear fluorescence intensity in old *C. neoformans* cells when compared to young *C. neoformans* (Figure 4A, *p* < 0.05). This increase in the DNA content was consistent with a prolongation in the G2 phase in old *C. neoformans* (Figure 4B, *p* < 0.001). However, no change in fungal ploidy was observed for both groups (Figure 4C).

In addition, we hypothesized the formation of the unbudded cells of old *C. neoformans* as a consequence of the delayed G2 phase. Indeed, only old-generation *C. neoformans* produced occasional yeast cells with morphological abnormalities, including cells of ellipsoidal shape, resembling a trimera yeast cell with two daughter cells formed from the same mother cell, or containing a granddaughter grown from the daughter cell (Figure 4D). These unusual tubular structures would suggest aberrant mitosis following the unbudded G2 arrest and reentry into the cell cycle. Taken together, these data indicate that aging has an effect on DNA quantity and the cell cycle, leading to a delay in the prolonged G2 phase with morphological abnormalities, such as trimera-like cell morphology. 

### 3.4. Vomocytosis Phenomenon during Replicative Aging in C. neoformans

**Figure 5:**
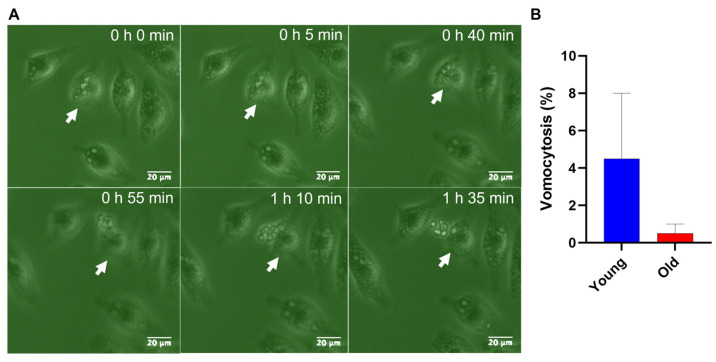
Vomocytosis events in macrophages (J774.A1) infected with young and old *C. neoformans* (H99 strain). (**A**) Representative real-time imaging of a vomocytosis type II event (white arrow) for macrophages infected with young *C. neoformans*. (**B**) Time-lapse microscopy videos were manually scored for the vomocytosis of *C. neoformans* from at least 100 macrophages containing yeast cells for each group until two hours after the phagocytosis assay. The graph shows the percentage of *C. neoformans*-infected macrophages which have experienced at least one vomocytosis event. Data from two independent experiments are shown. Categorical vomocytosis data were analyzed using a *t*-test followed by Welch’s correction (*p* = 0.455). Error bars represent the SEM.

Vomocytosis is a morphologically and temporally diverse process that occurs during macrophage infection [28]. We further explored the fate of young and old *C. neoformans* cells in the course of macrophage interactions with regard to vomocytosis (Figure 5A, Video S1). Vomocytosis can be classified as type I (complete emptying of macrophage) or II (partial emptying of macrophage) [28]. Macrophages infected with young *C. neoformans* experienced a higher rate of vomocytosis events when compared to macrophages infected with old *C. neoformans* (Figure 5B); however, this was not statistically supported, most likely due to vomycytosis being a rare event. All vomocytosis events for macrophages infected with old *C. neoformans* were type I, whereas 55% of vomocytosis events involving young *C. neoformans* ingested by macrophages were type II, and 45% were type I. All vomocytosis events observed for both groups were in non-acidic macrophages. These results indicate that younger *C. neoformans* cells are more likely to undergo non-lytic expulsion than old *C. neoformans*, and, interestingly, more than half of the non-lytic expulsions of young yeast cells were incomplete. 

Escape by macrophages can be influenced by the phagolysosomal pH [29]. Thus, we further analyzed the acidification of the host macrophages infected with young and old *C. neoformans*. 

### 3.5. Old C. neoformans Prefer to Reside in Acidified Phagosomes 

**Figure 6:**
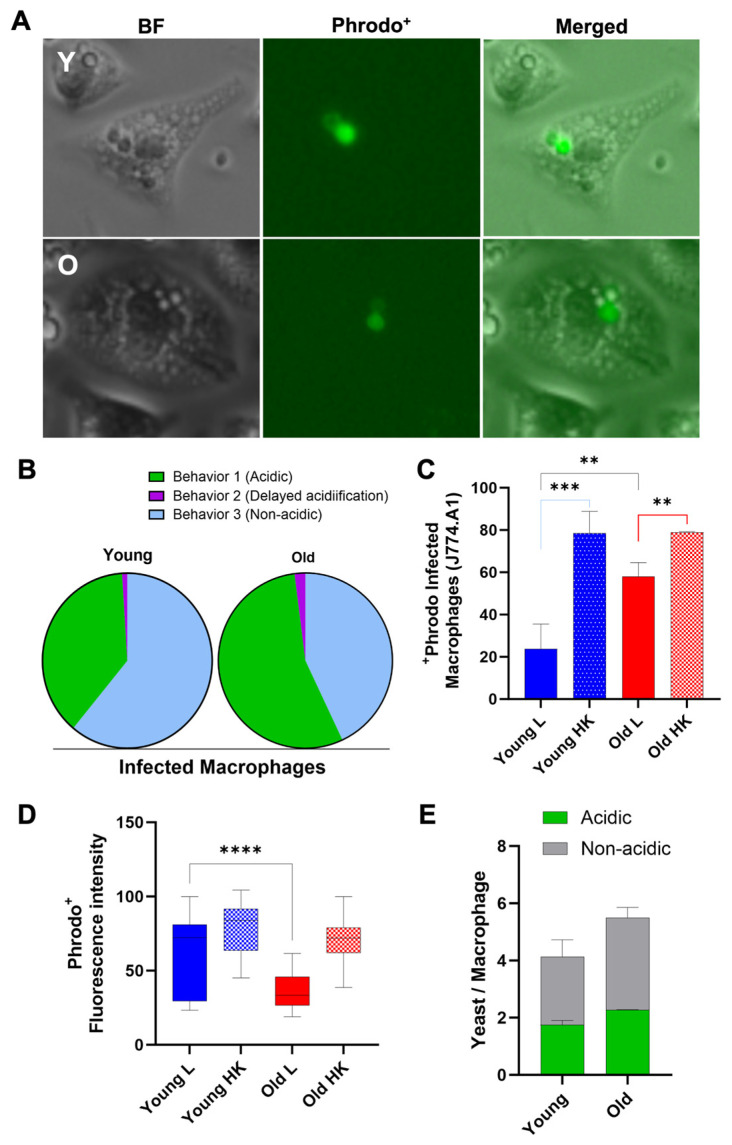
The effect of replicative aging on phagosomal maturation in macrophages containing H99 *C. neoformans*. (**A**) Represented image of the fluorescent microscopy of the macrophage mouse cell line J774.A1 infected with previously pHrodo-labeled cryptococci, both young and old (MOI of 10), after phagocytosis, showing acidified phagosomes. Scale bar represents 20 μm. (**B**) Macrophages were cultured with pHrodo-labeled live (L) cryptococci (young or old), or provided with the respective heat-killed (HK) yeast cells (positive control). Time-lapse microscopy videos two hours post-phagocytosis were manually scored for the percent change in pHrodo^+^ macrophages. At least 100 infected macrophages for each group were observed, and the statistical analysis of two independent experiments was performed using a *t*-test followed by post-Welch’s correction. The black line represents comparisons between young and old *C. neoformans*, the blue line between live and HK young *C. neoformans*, and the red line between live and HK old *C. neoformans* (** *p*< 0.01, *** *p* < 0.001). (**C**) Quantification of all behaviors of J774.A1 cells infected with *C. neoformans* (young and old) for up to two hours following phagocytosis. (**D**) Quantification of the Phrodo^+^ fluorescence intensity of macrophages ingested with either young (blue) or old (red), and their respective HK yeast cells (positive control). Images of at least 100 infected macrophages for each group were analyzed using ImageJ v.154g (NIH, Bethesda, MD, USA). Statistical analysis of two independent experiments was completed using an unpaired *t*-test and with post-Welch’s correction to compare macrophages ingested with live young against live old *C. neoformans* (**** *p* < 0.0001). (**E**) Quantification of the number of yeast *C. neoformans* cells per acidic (Phrodo^+^, green) or non-acidic (Phrodo^−^, gray) macrophages. At least 100 macrophages infected with young (Y) or old (O) *C. neoformans* were analyzed. Comparison between the groups of two independent experiments was performed using unpaired *t*-test and post-Welch’s correction (*p* = 0.2715).

Acidification is indicative of phagosomal maturation [15], and variations in the phagosomal acidic levels have been observed among macrophages infected with *C. neoformans* (Figure 6A). Here, we analyzed three distinct phagolysosomal response patterns: acidified phagolysomes (behavior 1), delayed acidification (behavior 2), and no acidification (behavior 3). For macrophages containing young *C. neoformans*, the most commonly (61%) observed behavior was the absence of acidification (behavior 3), whereas rapid acidification sustained up to 120 min (behavior 1) was found in 38% of macrophages infected with young cells. In contrast, only 43% of the macrophages containing old *C. neoformans* exhibited no acidification of phagolysosomes (behavior 3), whereas more than half (55%) had acidified phagolysosomes for up 120 min (behavior 1). Delayed acidification was uncommon after both the phagocytosis of young (2%) and old (1%) *C. neoformans* (Figure 6B,C). As expected, control experiments with heat-killed young or old *C. neoformans* confirmed that live fungus was required to modulate the phagolysosomal pH [30] (Figure 6B,D). When the mean fluorescence intensity (MFI) of only acidic phagosomes infected with either young or old *C. neoformans* was compared, MFI levels were found to be significantly higher for phagosomes containing young *C. neoformans* (*p* < 0.001, Figure 6D), consistent with a lower phagolysosomal pH. It is noteworthy that this difference was not caused by variations in the number of yeast cells per macrophage (Figure 6E). Taken together, these results indicate that, although the phagocytosis of old *C. neoformans* is more likely to lead to acidified phagolysosomes, the acidification is less pronounced than that of phagolysosomes containing young *C. neoformans*. The higher percentage of non-acidified phagolysosomes in macrophages infected with young *C. neoformans* was also associated with a higher number of vomocytosis events, which also included incomplete expulsions.

### 3.6. Generational Age in C. neoformans Influences Lysosomal Permeabilization

**Figure 7:**
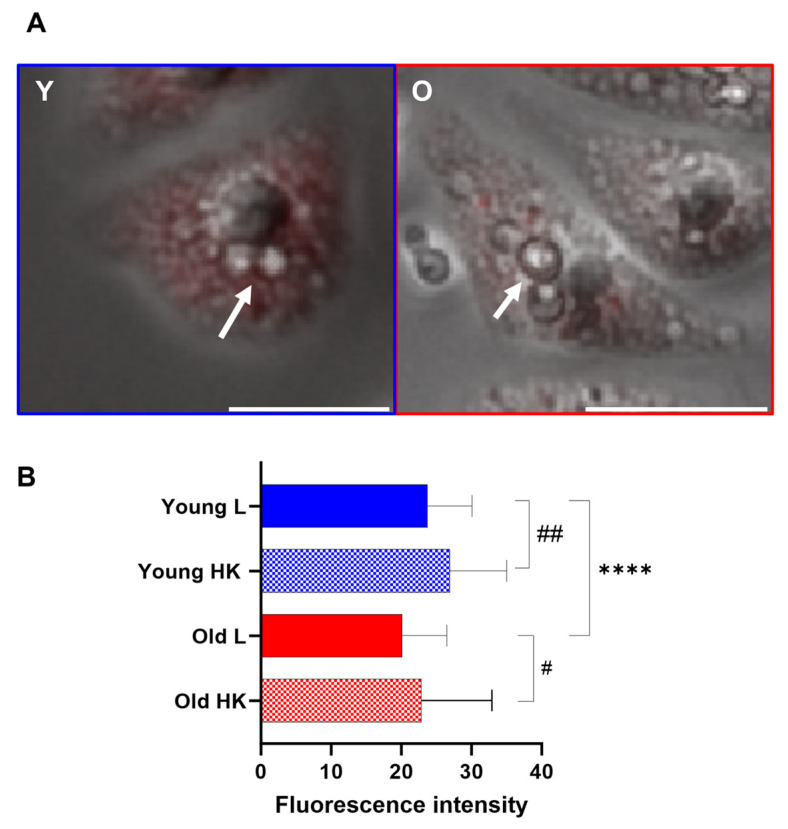
Phagosomal permeability of macrophages infected with H99 *C. neoformans*. (**A**) Representative images of fluorescent microscopy of macrophage mouse cell line J774.A1 stained with 50 nM LysoTracker^®^ Red DND-99 and previously infected with *C. neoformans* (white arrows), both young (Y) and old (O) (MOI of 10), showing phagosome leakage. Scale bar represents 25 μm. (**B**) The frequency of the phagosomal membrane permeability of macrophages infected with live (L) young (Y, blue) and old (O, red) *C. neoformans* and the respective heat-killed (HK) controls were measured using Lysotracker deep red staining. Macrophages with permeable phagosomes were determined via a loss of the Lysotracker deep red. The data shown are the quantification of the loss of the staining intensity, verified in 100 infected macrophages for each group, using images acquired following two hours of infection. Images were taken using Nikon Ti2-E PFS and analyzed using ImageJ v.154g (NIH, Bethesda, MD, USA). The bar chart shows the comparison of macrophages containing young with old *C. neoformans* (asterisk symbols) and live with HK *C. neoformans* (hashtag symbols). *p* values using a *t*-test with Welch’s correction (**** *p* < 0.001, # *p* < 0.05, ## *p* < 0.01).

We next analyzed the phagosome leakage as *C. neoformans* can manipulate phagosome acidification via permeabilizing the phagosome membrane [15]. Lysosome damage is crucial for intracellular *C. neoformans* survival strategy, and also contributes to fungal virulence [31]. Therefore, we assessed phagolysosomal permeabilization via real-time visualization and measuring LysoTracker DeepRed to intensity, localizing to the acidic organelle [12]. The number of cells presenting a loss of Lysotracker fluorescence was quantified following 2 h of infection. Macrophages infected with old *C*. *neoformans* developed significantly reduced Lysotracker fluorescence, indicating phagolysosomal membrane permeabilization. In contrast, macrophages infected with young *C. neoformans* retained higher levels of lysotracker fluorescence (Figure 7), suggesting the maintenance of phagolysosomal membrane integrity. As expected, the analysis of macrophages infected with heat-killed *C. neoformans* manifested no loss of fluorescence signal.

**Figure 8:**
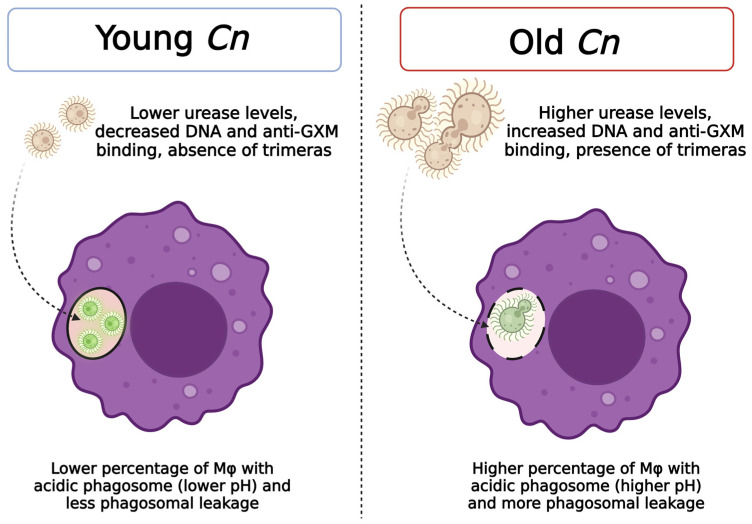
Schematic representation of replicative aging effects on the interactions of young and old *C. neoformans* (Cn) with host macrophage cells and fungal virulence-associated factors with intracellular persistence. This illustration was created using Biorender.

We synthesized the observations of this study in Figure 8.

## 4. Discussion

Interactions with macrophages play a crucial role during *C. neoformans* infection [32]. Phagosomal maturation involves fusion with lysosomes and subsequent acidification. This process is determined via phagosomal cargo, and is critical for the activation of mechanisms associated with antigen processing and presentation [33]. The survival of intracellular cryptococci despite the acidic luminal pH involves multiple antioxidant tools, such as the expression of enzymes, pigment production [34], and the production of capsular polysaccharide [30]. 

Previously, our lab showed that ten-generation old *C. neoformans* cells are significantly more resistant to killing by macrophages than their daughter cells [8,35]. However, major selection pressure would have to be operative in order to privilege these relatively rare old *C. neoformans* cells in the host environment. The accumulation of yeast cells with advanced generational age has been documented both for *C. neoformans* and *Candida glabrata* in several infection models [36,37]. The advanced generational age of *C. neoformans* cells is associated with increased melanin synthesis, reduced phagocytosis, and a thicker cell wall when compared to daughter cells [8,35]. Here, we report that old *C. neoformans* produce increased levels of urease and binding to capsular anti-GXM, as well as higher DNA content than young yeast cells. 

Urease activity leads to ammonia production and the elevation of phagosomal pH, since phagosomes containing *C. neoformans* (*ure1Δ*) and lacking urease activity presented a reduced pH when compared to macrophages loaded with the wild-type strain [12]. Our comparative analysis of phagosomal pH is compelling because, although a higher percentage of macrophages exhibit acidic phagosomes following the ingestion of old *C. neoformans* cells compared to those of phagocytose young *C. neoformans* cells, the pH of the acidic phagolysosomes is significantly lower when loaded with young *C. neoformans* (as shown in Figure 6C,D). Previous reports have shown that increasing the phagosomal pH with chloroquine is related to enhanced antifungal activity in macrophages infected with *C. neoformans* [38]. Thus, young cells mostly found in non-acidic phagosomal could indicate a better ability of the host cell to contain infections caused by young *C. neoformans*. In line with this finding, a lower pH was also associated with better *C. neoformans* replication [12]; therefore, we hypothesize that old cells may be using the macrophage as a reservoir in order to persist within the host cell under these conditions. Corroborating with our findings, it has previously been observed that an acidic pH generates a higher yield of dormant yeast cells [39]. Whether the differences in the phagosomal pH result in altered intracellular replication rates cannot be determined with the current experimental design, which was only able to image for up to 120 min. This technical limitation is because old *C. neoformans* cells would start replicating, generating a mixed population of younger daughter cells and older mother cells, which would make it difficult to differentiate the impact of aging. For the same reason, we did not assess intracellular replication rates. 

These data are consistent with published studies [15], which have shown that *C. neoformans* cells, when compared to other phagocytosed yeasts, has the unique ability to manipulate the acidification of their phagosomes. Studies with mutants have supported the concept that specific cryptococcal virulence factors, such as polysaccharide capsules and urease, determine the phagosomal dynamics. It is possible that the augmented excretion of urease into the phagolysosome via old *C. neoformans* cells can lead to a higher phagosomal pH. Additionally, the old cells may produce more polysaccharides and enhance the ability of ingested *C. neoformans* cells to better buffer phagosomal acidification [13]. The presence of GXM in vesicles [10] and the increased Golgi metabolism in old *C. neoformans* could be indicative of the production of more polysaccharide GXM. 

Our data demonstrate decreased lysotracker fluorescence in macrophages infected with old *C. neoformans*. The loss of acidity in phagosome could also be the result of enhanced phagosomal membrane leakage, which was documented in macrophages two hours following the phagocytosis of old yeast cells. The accumulation of infected macrophages with *C. neoformans* cells has been shown to lead to the rupture of the phagocytic cell [40]. Furthermore, the physical stress on membranes caused by capsular enlargement has also been linked to phagosomal leakage [13]. We have shown that aging results in cell enlargement [7], and that GXM-containing vesicles [10] and enhanced mAb anti-GXM binding to the capsule could be related to both the more pronounced loss of acidity, as well as the loss in phagosomal integrity in old cells. 

Furthermore, unbudded G2 arrest has been associated with changes in *C. neoformans* cell morphology, such as hyphal formation [41]. We observed trimera-like old *C. neoformans* cells, most likely because aging promotes G2 arrest. Since cell cycle arrest is also an important stress response mechanism in the murine pulmonary environment [14], we hypothesized that these alterations in the morphology of old cells could also contribute to the phagosomal integrity, resistance to phagocytic killing, and could even favor the rupture of the host phagocytic cells. Interestingly, *Candida albicans*, when exposed to fluconazole, forms three-lobed trimeras. These trimeras produce genetically variable progeny with varying numbers of chromosomes, thus increasing the odds of creating a drug-resistant strain [42]. Our analysis using flow cytometry for the DNA content of propidium iodide-stained cells could not identify aneuploidies. However, we hypothesize that old *C. neoformans* cells, when grown and isolated in the presence of fluconazole selection pressure, can produce more aneuploidies than young cells due to more genomic instability, which could cause more frequent gene copy number alterations.

Lastly, phagosome membrane permeabilization in macrophages infected with *C. neoformans* cells has been associated with a decrease in vomocytosis events [13], as well as a higher intracellular pH [25]. Non-lytic expulsion can be facilitated by non-acidic phagosomes [43], which could explain why we found more vomocytosis events in macrophages containing young *C. neoformans* cells than old cells, even though this was not a significant difference. Longer live-imaging videos would likely have captured more vomocytosis events; the majority of *C. neoformans* cells are reported to exit via non-lytic expulsion after six hours [28]. The prolonged imaging and analysis of more infected phagocytic cells would also clarify if dragatocytosis (lateral transfer from one macrophage to subjacent macrophage) is enhanced in macrophages infected with old *C. neoformans* cells. Previous studies have demonstrated that the actin flash formation may be a macrophage mechanism to avoid *C. neoformans* cell escape via vomocytosis [25]. Given that the size, capsule, and cell wall is markedly altered in old *C. neoformans* cells, it is conceivable that the actin flash formation also differs in macrophages infected with old *C. neoformans* cells when compared to young *C. neoformans* cells. 

Taken together, our data suggest that the aging of *C. neoformans* cells results in altered interactions with the phagolysosomes in macrophages, which supports our hypothesis that macrophages play a key role in the selection process of old *C. neoformans* cells during infection. Old *C. neoformans* cells prevail within acidic phagolysosomes and manipulate the phagosomal pH, both of which are consistent with an enhanced resistance to macrophage killing. Future experiments employing prolonged live imaging studies and aged urease and capsular mutants will help to further elucidate the effect of old *C. neoformans* cells on phagosomal interactions and intracellular fate. The comprehension of these essential host–pathogen interactions could further shed light on mechanisms that bring about new insights for novel antifungal therapeutic designs.

## Figures and Tables

**Table 1:** Golgi morphologies pattern in young and old *C. neoformans*.

Golgi Morphology	Young	Old
Peripherical	62.85%	60%
Central	37.15%	40%

## Data Availability

All data required to understand this article are presented in the study. Any raw data further requests will be provided by the corresponding authors.

## Supplementary Materials

The following supporting information can be downloaded at: https://www.mdpi.com/article/10.3390/jof10040279/s1, Video S1: Vomocytosis event in macrophage infected with *C. neoformans*.
